# A Data-Centric Diagnostic Framework for Experimental Design and Mechanistic Modeling: Application to mRNA Production

**DOI:** 10.1021/acs.iecr.6c02860

**Published:** 2026-10-01

**Authors:** Ioanna Kalospyrou, Farshid Babaei, Erly Sintya, Zoltán Kis, Joan Cordiner, Solomon Brown

**Affiliations:** † School of Chemical, Materials and Biological Engineering, 7315 The University of Sheffield, Sheffield S1 3JD, U.K.; ‡ Warmadewa Research Centre, 202244 Universitas Warmadewa, Denpasar, Bali 80235, Indonesia

## Abstract

In this study, we present a data-centric framework for the integrated investigation of experimental behavior and mechanistic model performance in complex biochemical systems. The framework is demonstrated on the *in vitro* transcription (IVT) reaction for mRNA production. Rather than analyzing experimental data and mechanistic models independently, the framework links experimental-response surrogate modeling, a surrogate model of error characterizing model–experiment discrepancy, and mechanistic parameter sensitivity within a unified workflow. Gaussian Process Regression (GPR) and Global Sensitivity Analysis (GSA) are first used to interrogate experimental data, identify statistically anomalous observations, and characterize the process variables and interactions governing the response. Predictions from the resulting experimental-response surrogate are then compared with those of an existing mechanistic model, and the resulting discrepancy is represented through a surrogate model of error and subjected to GSA. Finally, the process-level drivers of this discrepancy are cross-compared with sensitivities of the mechanistic kinetic parameters, thereby linking experimental and mechanistic information to prioritize subsequent investigation. The experimental-response surrogate achieved an *R* ^2^ of 0.90, with MgOAc and NTP concentrations dominating variation in mRNA yield and incubation time contributing principally through interactions. The surrogate model of error achieved an *R* ^2^ of 0.86 and identified NTP, MgOAc, T7-RNA polymerase, and selected interactions as the principal contributors to the discrepancy between mechanistic and experimental-surrogate predictions. Cross-comparison with mechanistic parameter sensitivities subsequently prioritized the Mg degradation rate constant (*k*_*Mg*_), DNA equilibrium constant (*K*_*DNA*_), Mg saturation constant (*k*_1_), and transcription rate constant (*k*_*app*_), together with their associated model components, for further investigation. The resulting framework provides a systematic basis for directing subsequent repeat or targeted experimentation, parameter recalibration, and structural model refinement.

## Graphical abstract

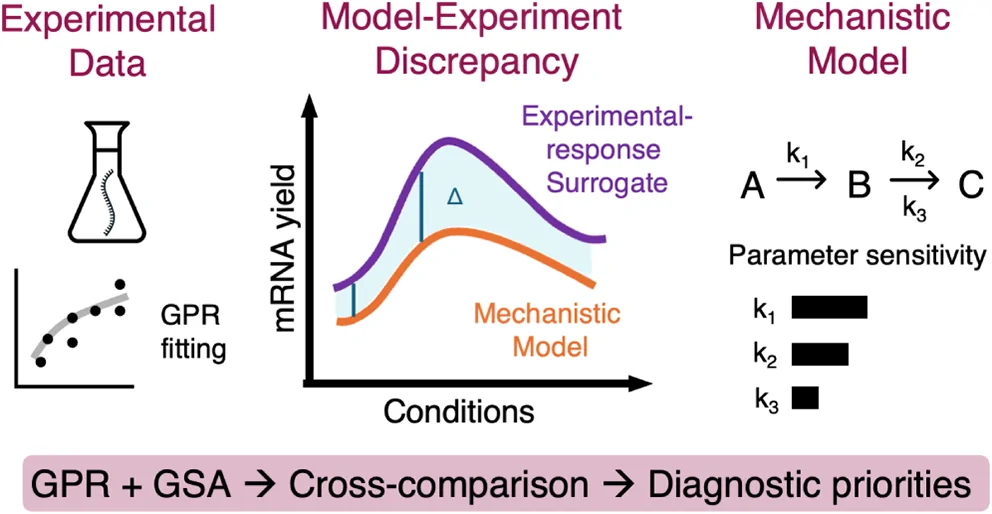

## 1 Introduction

The global rollout of mRNA vaccines during the COVID-19 crisis showcased their potential in both preventive and therapeutic medicine. Their rapid development and deployment in 2020 paved the way for deeper exploration into mRNA as a transformative biomedical platform. Therapeutic mRNA technologies, particularly those enabling targeted protein delivery to specific organs,^1^ now stand at the forefront of treatment innovation for diseases previously considered intractable.^2^ With over 100 clinical trials underway, largely focused on infectious diseases and cancer, the mRNA market is projected to surge to $25 billion by 2030.^2^ Nonetheless, the scalability of mRNA therapies is constrained by high production costs, driven by approximately 15 operational stages including plasmid production, *in vitro* transcription (IVT) reaction, mRNA purification, and lipid nanoparticle encapsulation. Among these, the IVT process accounts for the highest cost, largely due to the demand for specialized enzymes and capping analogs.^2^ Addressing these financial bottlenecks while maintaining high product quality and homogeneity hinges on a thorough understanding of IVT encompassing reaction kinetics and dynamics. Although several efforts have aimed at such a goal through isolated empirical tweaks or theoretical modeling, the field still lacks a synergistic framework that harmonizes experimental insights with a mechanistic understanding.

IVT systems enable the study of gene expression dynamics in controlled environments without the complexity of living cells.^3^ A brief explanation of the full mechanism is presented in Figure 1.

**Figure 1:**
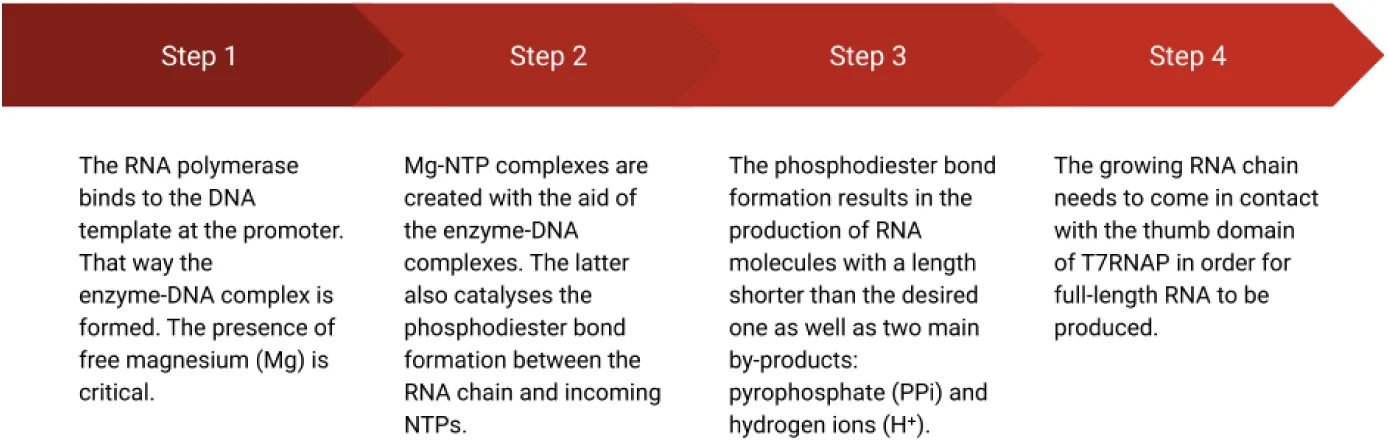
mRNA *In Vitro* Transcription Mechanism.^4,5^

Transcription is generally the process of encoding a certain region of the DNA template into messenger RNA (mRNA). For mass production of mRNA, transcription is an *in vitro*, cell-free process which takes place in the aforementioned bioreactor. The reaction is facilitated by certain enzymes called bacteriophage RNA polymerases and it takes place in a buffer solution whose most crucial component is magnesium.^6^ NTPs are used as the raw materials, substrate for the enzymes.

Due to the tightly coupled nature of transcription and translation, resource constraints, and the interplay between deterministic and stochastic dynamics, parameterization is both essential and inherently difficult.^7^ Accurate parameterization enables predictive modeling, mechanistic insight, and robust scenario analysis, yet biological systems are rife with variability across scales with key parameters such as transcription rates fluctuating between batches or even within single-cell populations. Many of these parameters are not directly observable and must be inferred from sparse or noisy data, introducing uncertainty and potential bias. Moreover, stochastic behaviors such as transcriptional bursting and translational pausing demand distributional estimates rather than fixed values, complicating inference and requiring richer datasets and advanced techniques. Resource limitations further introduce nonlinear feedbacks that are highly sensitive to parameter values, and mis-specification can undermine model reliability, scalability, and integrity. Thus, while parameterization underpins the credibility and utility of transcription models, it demands iterative refinement, sensitivity analysis, and a careful balance between empirical rigor and biological plausibility.^8^

Since empirical optimization of IVT is resource-intensive and slow to adapt to changing conditions, modeling approaches from simple black-box models to sophisticated mechanistic approaches have been utilized.^3^ As the predominant approach, ordinary differential equation (ODE)-based models typically entail mass action kinetics to describe the interactions between molecular components. Probabilistic state-based models have been proposed for kinetic parameter estimation,^9^ mass-action ODE frameworks with structural identifiability analysis^7^ and coupled ODE biophysical models integrating literature parameters with experimental data via multi-objective optimization.^10^ Similar modeling paradigms have also been used to capture gene expression dynamics across bacterial and mammalian systems^11,12^ predict IVT performance,^13^ and guide RNA vaccine production.^14,15^

Additionally, Bayesian inference methods have been employed to address parameter uncertainty and incorporate prior knowledge into the estimation process.^16^ This framework enabled inference of previously unknown transcription factor binding affinities and quantification of resource sharing in cell-free systems. Furthermore, Rosa et al. applied the same technique to optimize reaction yield and experimental settings of mRNA vaccine production.^8^ An alternative approach involves constraint-based strategies that model transcription-translation processes, potentially avoiding the need for detailed kinetic parameters. Dai et al. developed dynamic constraint-based modeling approaches for cell-free protein synthesis that incorporated metabolite measurements directly into flux estimation problems.^17^ Vilkhovoy et al. demonstrated that sequence-specific constraint-based modeling could explicitly couple transcription and translation processes with metabolic resource availability.^18^

Despite these methodological advances, to the best of authors’ knowledge, the IVT process as a highly complex biochemical mechanism has not yet been comprehensively understood. Parameterization remains problematic, with many kinetic parameters often difficult to identify.^17^ Moreover, the quality of available evidence varies considerably, as many studies rely on limited experimental datasets or prioritize model development over rigorous validation. Progress toward a holistic framework will require high-quality data integration, robust outlier detection, and the combination of artificial intelligence (AI) with mechanistic modeling to uncover process dynamics, critical parameters, and their interactions.

The current study focuses on the upstream process of an mRNA production line, specifically targeting the batch IVT reactor. The main contribution is a data-centric computational framework that integrates experimental and mechanistic model investigation through the sequential use of GPR and GSA. Rather than analyzing experimental data and mechanistic model behavior independently, the framework links information obtained across three stages:

- Stage I – Experimental data diagnosis: GPR-based analysis is used to identify statistically anomalous experimental observations and prioritise conditions for further experimental reassessment.
- Stage II – Experimental response characterization: GSA of the experimental-response surrogate is used to identify the process variables and interactions governing variation in mRNA yield within the investigated operating space.
- Stage III – Mechanistic model diagnosis: the discrepancy between experimental-surrogate and mechanistic-model predictions is characterized and its process-level drivers are cross-compared with mechanistic parameter sensitivities to prioritize candidate parameters and model components for further investigation.

The resulting framework therefore enables experimental behavior and mechanistic model performance to be interrogated within a common data-centric workflow, with the model–experiment discrepancy acting as a link between process-level and mechanistic information. The resulting diagnostic outputs can subsequently inform repeat or targeted experimentation, parameter recalibration, and structural model refinement, which constitute feedback actions for further iterations of the framework.

The remainder of this study is structured as follows: Section 2 outlines the underlying principles of GPR and the methodological approach employed; Section 3 defines the case study used; Section 4 details the results alongside a comprehensive discussion of their implications; and Section 5 concludes the study with a synthesis of key findings and potential directions for future research.

## 2 Methodology

### 2.1 Background

The selection of a data-driven or surrogate-assisted methodology depends strongly on the intended analytical objective, which may include process monitoring, targeted optimization, experimental design, parameter estimation, or global investigation of model behavior.^19–21^ The present study adopts a combination of Gaussian Process Regression (GPR) and Global Sensitivity Analysis (GSA) to support the integrated investigation of experimental behavior and mechanistic model performance. Both analyses are implemented using the rom-comma software package,^22^ which provides Gaussian process surrogate modeling and variance-based GSA. The principles of these two methods are introduced below before their combined role within the proposed framework is discussed.

GPR is a nonparametric Bayesian approach to regression problems widely used in machine learning, through which, an extensive range of relationships between inputs and outputs can be captured. This is achieved through the utilization of an infinite number of parameters and allowing for the data to determine the level of complexity through Bayesian inference. The implementation of GPR necessitates the selection of a kernel function, k, the purpose of which is to express the covariance between the outputs, y­(x) and y­(x′), observed at different inputs, x and x′ as presented in eq 1.

1 $$k\left(x,x^{\prime }\right)=Cov\left(y\left(x\right),y\left(x^{\prime }\right)\right)$$

Where x and x′ are vectors in Euclidean space. Namely, the kernel function measures the similarity of two data points, x and x′, in the input space. There are multiple types of kernels, yet the most widely used one and selected in this study is the Radial Basis Function (RBF). Its formula is presented in eq 2 where *k*_*SE*_ symbolizes the kernel function, x and x′ are two random data points and *l* and *σ* are the length and output scales, respectively.

2 $$k_{SE}\left(x,x^{\prime }\right)=\sigma^{2}\exp\left(-\frac{({x-x^{\prime })}^{2}}{2l^{2}}\right)$$

The length and output scales are two of the hyperparameters that need to be tuned in a kernel for it to effectively measure the similarity of the corresponding function values.

Sensitivity Analysis (SA) is defined as “the study of how uncertainty in the output of a model (numerical or otherwise) can be apportioned to different sources of uncertainty in the model input”.^23^ SA exploits the “sparsity of factors” principle, which states that only a small subset of factors in a system are statistically significant to the system output. GSA, rather than local sensitivity methods, evaluates the influence of model inputs across the defined input space. Variance-based GSA therefore provides a systematic means of identifying influential variables and interactions and quantifying their contributions to variation in the modeled response.

The combination of GPR and GSA adopted in this study is particularly suited to the global diagnostic objective of the proposed framework. Multivariate chemometric techniques such as Principal Component Analysis (PCA) and Partial Least Squares (PLS) have historically been widely applied for process diagnostics and multivariate monitoring in chemical and bioprocess systems.^24,25^ Their predominantly linear latent-variable representations, however, can limit the representation of strongly nonlinear relationships and higher-order interactions unless suitable nonlinear extensions are introduced. This is particularly relevant for complex biochemical systems, such as IVT, in which interactions between reaction components and operating conditions may strongly influence the observed response.

Adaptive methodologies such as Bayesian Optimization (BO) and Active Learning (AL) address a different analytical objective by selecting new observations sequentially according to an acquisition criterion, for example to improve an optimum or reduce predictive uncertainty. Such methods can be highly effective for targeted optimization and experimental design, but their deliberately nonuniform sampling can leave regions that are less relevant to the acquisition objective comparatively under-represented.^26,27^ This differs from the objective of the present framework, where the aim is to interrogate variation across a predefined operating space and determine which variables and interactions contribute most strongly to the experimental response and to model–experiment discrepancy.

GPR provides a flexible nonparametric representation of potentially nonlinear input–output relationships and an associated representation of predictive uncertainty, while GSA decomposes output variability across the defined input space.^19^ Their combination therefore enables influential variables and interactions to be identified without restricting the analysis to behavior around a single operating point. Within the proposed framework, the same analytical principle is applied across the experimental-response surrogate, the surrogate model of error, and the mechanistic parameter space. This provides a common quantitative basis through which experimental response sensitivities, process-level drivers of model–experiment discrepancy, and mechanistic parameter sensitivities can subsequently be cross-compared.

Importantly, the purpose of this analysis is not to establish parameter identifiability or to demonstrate that particular mechanistic equations are structurally incorrect. Rather, the combined GPR–GSA analysis is used to characterize variation and discrepancy across the investigated space and to prioritize experimental conditions, process variables, kinetic parameters, and associated model components for subsequent investigation.

Operationally, the variance-based GSA component of the framework decomposes the output variance into contributions attributable to individual inputs and groups of inputs. The framework was set by Ilya Sobol^28^ and it is based on the Sobol sensitivity indices which measure the degree of significance to which the variance of different model parameters affects the model output. The GSA results from the package comprise three different types of Sobol indices: First Order, Closed and Total indices. Definitions of different types of Sobol indices, particularly closed ones, are not very consistent across literature.^29,30^ A schematic representation of these relationships is presented in Figure 2.

**Figure 2:**
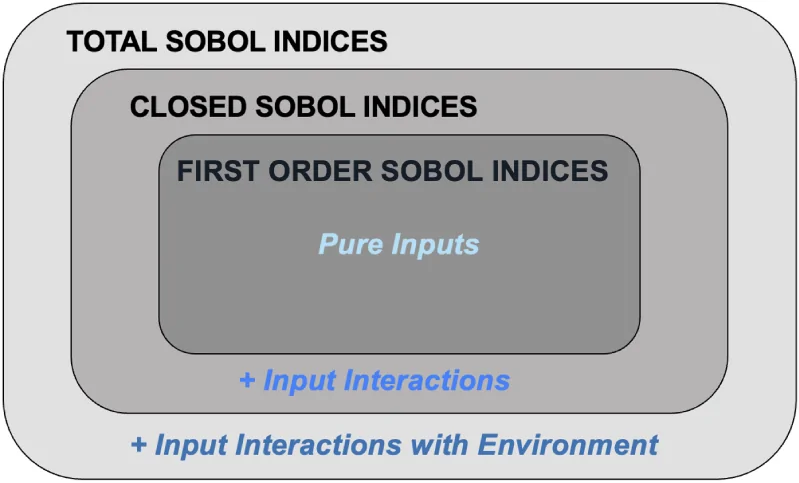
Relationship between different types of Sobol indices and what they include.

As depicted in Figure 2, the closed index of a set of inputs showcases the statistical significance of all input parameters and their interactions on the output variance. The first order index is defined as the closed index of a single input. Namely, it will showcase the statistical significance of each input on the output with no parameter interactions considered. Finally, the total index of a set of inputs includes all interactions of the set with any input even outside the closed system of inputs examined.

Consequently, the two key Sobol indices generated are the First Order and Closed ones, since those two provide information solely on the specified set of inputs. Moreover, through simple subtractions between the closed and first order results, the most critical interactions between the system components can easily be identified.

### 2.2 Proposed Workflow

The proposed methodology provides a data-centric framework for the integrated investigation of experimental behavior and mechanistic model performance. Rather than treating experimental analysis and mechanistic modeling as independent activities, the framework progressively links information obtained from experimental-based modeling, model–experiment discrepancy analysis, and mechanistic parameter sensitivity. This approach is particularly relevant to systems for which experimental data are available alongside a mechanistic representation, but where the underlying mechanism remains incompletely understood or discrepancies between experimental and modeled behavior persist. The proposed workflow is illustrated in Figure 3.

**Figure 3:**
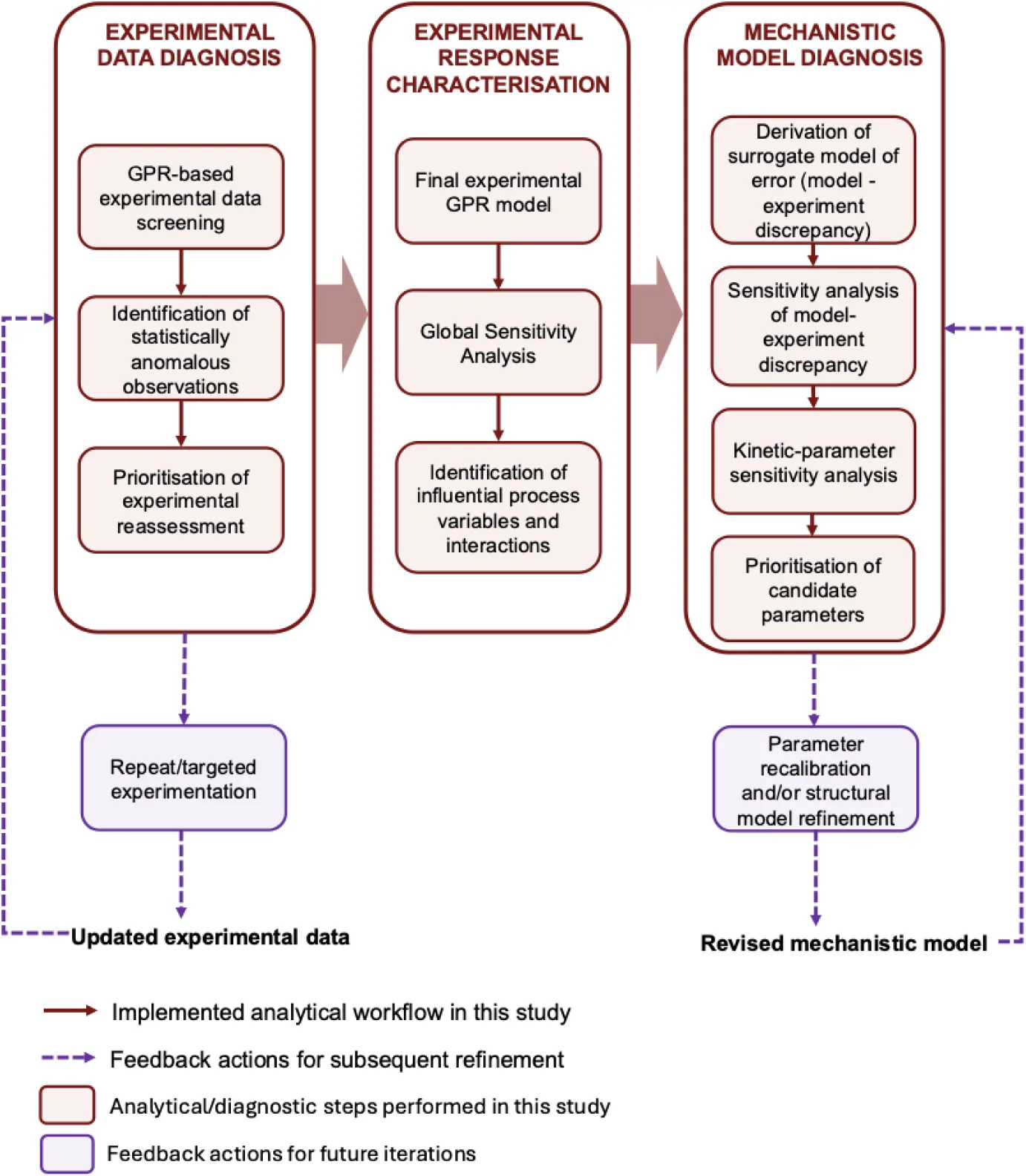
Step-wise schematic representation of the proposed data-centric framework for integrated experimental and mechanistic model investigation. Solid pathways denote the analytical and diagnostic steps implemented in the present study, whereas dashed pathways denote subsequent feedback actions through which the identified experimental conditions and mechanistic model components can inform repeat or targeted experimentation, parameter recalibration, and/or structural model refinement.

As shown in Figure 3, the implemented workflow consists of three interconnected stages:

1 **Experimental data diagnosis**, in which GPR-based screening is used to identify statistically anomalous observations and prioritise experimental conditions for reassessment;
2 **Experimental response characterization**, in which GSA of the final GPR model fitted to the retained experimental data, hereafter termed the experimental-response surrogate, is used to identify the process variables and interactions governing variation in the experimental response;
3 **Mechanistic model diagnosis**, in which the discrepancy between predictions from the experimental-response surrogate and the mechanistic model is represented using a second GPR model, termed the surrogate model of error, and its process-level sensitivities are cross-compared with mechanistic kinetic-parameter sensitivities to prioritize candidate parameters and model components for further investigation.

The framework additionally defines feedback pathways through which the outputs of these analyses can inform subsequent corrective actions. Experimental conditions prioritized for reassessment may motivate repeat or targeted experimentation, generating updated experimental data that can be reintroduced into the workflow. Similarly, prioritized mechanistic parameters and model components may motivate parameter recalibration and/or structural model refinement, after which the revised mechanistic model can be reassessed against the experimental representation. These feedback actions are not implemented in the present case study and are therefore distinguished from the demonstrated analytical workflow in Figure 3.

The computational structure of the framework is not specific to a particular biochemical reaction mechanism. GPR-based surrogate modeling and variance-based GSA provide general tools for identifying influential variables and parameters in complex systems,^19^ while integration of data-driven and mechanistic representations has been increasingly used to support the analysis and refinement of chemical and biochemical process models.^20^ Nevertheless, interpretation of the resulting sensitivities and model–experiment discrepancies remains dependent on the physical and biochemical context of the system under investigation. Accordingly, the outputs of the present framework are treated as diagnostic evidence used to prioritize subsequent investigation rather than as formal validation of individual mechanistic equations.

### 2.3 Experimental Data Processing

The proposed workflow starts with sampling the experimental input space using a Latin Hypercube Sampling (LHS) scheme. LHS provides uniform coverage across all dimensions of the design space, ensuring that the resulting dataset is sufficiently rich for surrogate modeling. These sampled input combinations are then used to conduct controlled experimental runs, forming the basis for subsequent GPR fitting and analysis within the rom-comma framework. The first fitting of experimental data to the GPR model triggers the data cleaning process. Within the rom-comma package, output normalization assumes a Gaussian distribution, allowing for both positive and negative values. When the measured response is strictly non-negative, this assumption leads to infeasible negative predictions. To mitigate this, a logarithmic transformation of the response is applied as the GPR model output.

Once the experimental data are fitted with GPR, the predicted values generated for the different sets of input conditions are compared to the actual output values obtained experimentally for those conditions. After GPR fitting, the software provides the Z-score, a standard score representing the deviation of experimentally obtained yields from GPR-predicted values, divided by the standard deviation of the GPR curve at that point. This standardization expresses how far each data point lies from the mean in units of standard deviation. Under the assumption of normality, 99.7% of data should fall within ±3 standard deviations. Therefore, any condition set with a Z-score beyond ±3 is identified as statistically anomalous relative to the fitted GPR model. While typical strategies for handling outliers include replacing them with the mean or median of nonoutlying data, in this case, all condition sets with outlying results are removed from the dataset. Importantly, identification as statistically anomalous does not by itself establish that an observation results from experimental error, as such behavior may also reflect experimental variability or process behavior not adequately represented by the surrounding observations.

Within the proposed framework, these conditions are therefore prioritized for repeat or targeted experimentation. The resulting observations could subsequently be incorporated into an updated experimental dataset and reassessed through a further iteration of the workflow. This feedback step is not implemented in the present case study.

The resulting experimental-based GPR model constitutes the experimental-response surrogate. A GSA is then conducted on this surrogate to determine which input parameters and interactions, based on the first-order and closed Sobol indices, contribute most strongly to variation in the output.

### 2.4 Surrogate Model of Error between Mechanistic Model- and Experimental-Based Predictions

We use LHS to generate mechanistic model predictions because it preserves each variable’s marginal distribution while respecting target inter-variable correlations.^31^ This space-filling design enables accurate response estimation and efficient definition of the design space with relatively few samples.

A set consisting of some condition samples is generated and fed into the mechanistic model-based simulation and the experimental-based GPR model generated (see Section 2.3) to make predictions for the outputs. Based on the predictions obtained from both models, the logarithmic residual between the predictions for every set of conditions is determined as presented in eq 3.

3 Residual=ln(simulation response)−ln(experimental‐based GPR response)

A new GPR model is then constructed using input variables, with the model output defined as the residual associated with each set of experimental conditions. This model is termed the surrogate model of error and depicts the error between the simulation results and the experimental-based predictions. A GSA is then conducted on this model to determine the key inputs influencing this error most.

### 2.5 Mechanistic Model: Focus on Influential Parameters

To further examine the influence of individual parameters on mechanistic model predictions of outputs, a GPR model is constructed using varying parameter values as inputs and the predicted response as outputs. A GSA is then performed on this GPR model, focusing exclusively on first-order Sobol indices due to the absence of interactions among the parameters. The resulting sensitivity rankings are cross-compared with the process-level drivers identified through the model–experiment discrepancy analysis. Consistency between these analyses is used to prioritize kinetic parameters and associated model components for further mechanistic investigation. This analysis does not establish parameter misspecification or structural inadequacy of individual mechanistic equations; confirmation of either would require subsequent parameter recalibration or model reformulation followed by reassessment against experimental observations.

## 3 Case Study

To describe and predict the outcomes of a batch IVT reactor system, the mechanistic IVT modeling frameworks of van de Berg et al.^14^ and Ahmed et al.^15^ are considered. These works provide comprehensive mechanistic and process-level representations of IVT reactor behavior suitable for quantitative analysis and optimization.

The experimental dataset utilized in this work originates from the IVT experiments reported in ref 15 involving operation of a 100*μ*L batch IVT reactor under varying operating conditions. Detailed descriptions of the experimental setup, analytical procedures, and data generation methodology are provided in the original study, while associated IVT analytical and sample quantification methodologies are further described elsewhere.^32^ The dataset comprises 60 experiments with 2 replicates each.

The conditions varied within the IVT reactor and the ones that remain constant in this experimental setup are provided in Tables 1 and 2, respectively:

**Table 1:** Varied Parameters in Experiments

Parameter	Range	Units
Nucleoside Triphosphate (NTP) Concentration (individual)	1–15	mM
Magnesium Acetate (MgOAc) Concentration	6–90	mM
T7-RNA Polymerase Concentration	100–400	U/*μ*L
Template DNA Concentration	20–100	*μ*g/mL
Spermidine Concentration	1–4	mM
Incubation Time	0.25–4	hr

**Table 2:** Constant Parameters in Experiments

Parameter	Value	Units
Temperature	37	^*o*^C
pH	7.5	-
Dithiothreitol (DTT) Concentration	10	mM
Inorganic Pyrophosphatase (IPP) Concentration	0.05	U/*μ*L
Sodium Chloride (NaCl) Concentration	50	mM
HEPES Concentration	40	mM
RNAse Inhibitor Concentration	1	U/*μ*L

As noted before, since mRNA yield is strictly non-negative, the natural logarithm of the mRNA yield is used as the GPR model output. Following the methodology workflow, once the experimental data is cleaned, a final GPR model is constructed and subjected to GSA. First–order and total–effect Sobol indices are used to identify the most influential input parameters and interactions governing the mRNA yield.

In line with the second stage (Section 2.4), a set of 900 condition samples is generated and supplied to both the mechanistic model-based simulation and the experimental-based GPR model to obtain predictions for the mRNA yield. The logarithmic residual between the two model predictions is then calculated for each condition set, following the previously defined eq 3, but now applied with the response interpreted as yield. At this stage, the surrogate model of error is generated with inputs being the NTP, MgOAc, T7-RNA Polymerase, template DNA, spermidine concentrations and incubation time and output being the residual (see eq 3) for each set of samples.

Toward the final step (see Section 2.5), the original mechanistic model of van de Berg et al.^14^ includes a set of kinetic parameters, which are summarized along with their descriptions in Table 3. Some of these parameters can be tuned to maximize the efficiency of the model without limitations while others need to be within reason from a thermodynamic and chemical kinetic standpoint (e.g., equilibrium constants).

**Table 3:** Kinetic Parameters in the Mechanistic Model^14^

Symbol	Description
*k*_*d*_	T7-RNAP inactivation rate constant
*k*_*DNA*_	DNA equilibrium constant
*k*_*app*_	Transcription rate constant
*k*_1_	Mg saturation constant
*k*_2_	MgNTP saturation constant
*k*_*ac*_	Acidic degradation rate constant
*k*_*ba*_	Basic degradation rate constant
*k*_*Mg*_	Mg degradation rate constant

Using the procedure described in Section 2.5, a GPR model is built from sampled kinetic constants and their predicted yields, and a first-order Sobol GSA is performed. The resulting sensitivity rankings either reinforce the experimental-based GPR results or indicate possible issues in the underlying mechanistic equations.

For the dataset sizes considered in the present study, the GPR–GSA stages of the workflow are computationally modest. For the experimental analysis, comprising 120 observations and six input dimensions, 10-fold GPR processing using rom-comma was completed on the order of seconds on an Intel­(R) Core­(TM) Ultra 5 135H (3.60 GHz) processor and 16GB RAM. Following surrogate fitting, the Sobol sensitivity indices are evaluated semi-analytically from the fitted Gaussian process, avoiding the need for extensive additional sampling of the underlying model.^22^ Computational requirements increase with dataset size and input dimensionality through the increased cost of GPR fitting and sensitivity evaluation. Nevertheless, rom-comma-based GPR–GSA analysis has previously been demonstrated with substantially larger training datasets, including 2,745 observations with five input dimensions and 5-fold cross-validation,^33^ indicating applicability to dataset sizes considerably larger than those considered here. The computational cost associated with generating mechanistic-model predictions is application-specific and depends on the external mechanistic model and numerical solver rather than on the GPR–GSA analysis itself.

## 4 Results and Discussion

This section presents results generated from the application of the computational methodology on the mRNA IVT process. In the documented results, the statistical significance always corresponds to the Sobol sensitivity index contribution to output variance.

### 4.1 Experimental Data Cleaning: Outlier Determination

The initial experimental dataset comprised 120 individual observations, corresponding to 60 experimental conditions measured in duplicate. The experimental data cleaning procedure was performed iteratively over nine GPR fitting steps. At each step, observations falling outside the defined Z-score threshold were identified, and the remaining observations were used to refit the GPR model. Across the complete procedure, 26 of the 120 observations (21.7%) were identified as statistically anomalous, leaving 94 observations (78.3%) in the final experimental dataset. Importantly, observations were identified exclusively according to the GPR-based statistical screening criterion rather than on the basis of their mRNA yield, incubation time, or MgOAc-to-NTP ratio. A GSA was additionally performed following each GPR fit, with NTP and MgOAc concentrations consistently identified as the principal contributors to output variance across the successive fitting steps.

Based on prior observations (refs 34 and 35), the MgOAc-to-total-NTP ratio associated with high mRNA yields lies approximately within the range of 1–1.9. Following the GPR-based statistical screening, the observations identified as statistically anomalous were subsequently examined in relation to this ratio to explore whether any common process characteristics were evident. Figure 4 therefore presents the experimental yields of these observations against the MgOAc-to-total-NTP ratio. Importantly, the ratio itself was not used as a criterion for identifying or removing observations during the data-cleaning procedure.

**Figure 4:**
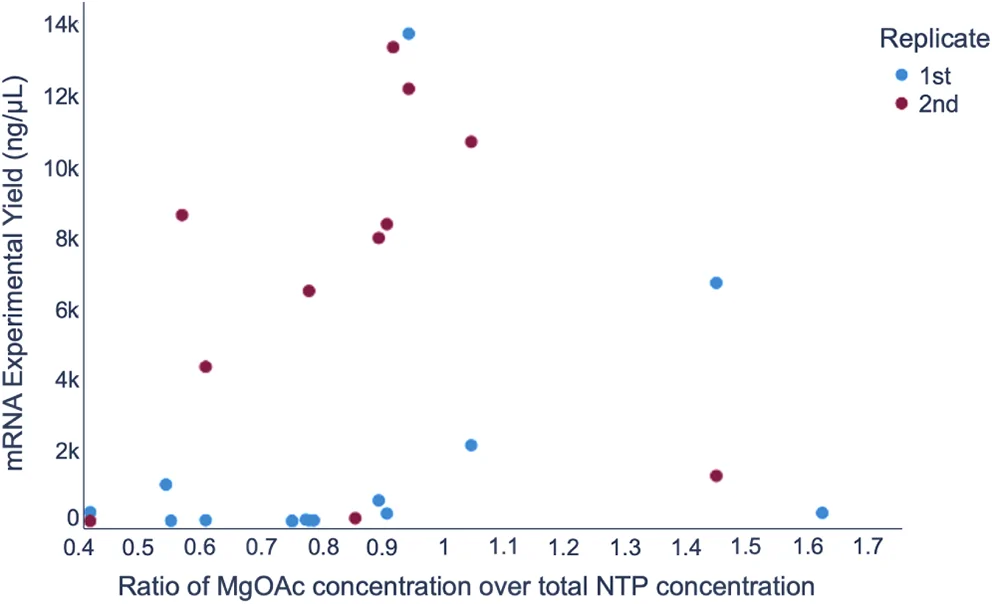
Experimental data points from experiments removed during the cleaning process.

Among the observations identified as statistically anomalous in the first experimental replicate, several exhibit very low mRNA product generation, as presented in Figure 4. Many of these observations occur at MgOAc-to-total-NTP ratios that are not expected to maximize mRNA production, although sufficient NTP and MgOAc are present for the IVT reaction to proceed. These observations may therefore reflect experimental variability or systematic experimental error, but may also represent reproducible process behavior under less favorable IVT conditions. In particular, low mRNA yield is not itself treated as evidence of experimental error. The statistical screening alone cannot establish the origin of these observations. Within the proposed framework, these conditions are therefore prioritized for repeat or targeted experimentation to distinguish experimental variability from reproducible process behavior.

Among the observations identified as statistically anomalous in the second experimental replicate, some similarly exhibit low mRNA yields, whereas others exhibit comparatively high yields at MgOAc-to-total-NTP ratios outside the range generally associated with high mRNA production. These observations differ from the dominant behavior of the experimental dataset and from trends reported in previous studies, and are therefore likewise prioritized for further experimental investigation.

Finally, for some experimental conditions, observations from both replicates were identified as statistically anomalous during the screening procedure. These conditions generally occur at MgOAc-to-total-NTP ratios below 1.1 and exhibit substantial variability in the measured mRNA yields, which may indicate increased process variability or instability under these conditions.

To assess whether filtering materially affected the experimental-response surrogate, the initial model fitted to all 120 observations was compared with the final model fitted to the 94 retained observations. Model performance was assessed using 10-fold cross-validation, distinct from the nine sequential filtering and refitting steps described above. Mean *R* ^2^ increased from 0.52 to 0.90, while mean root mean squared error (RMSE) decreased from 0.67 to 0.26 and mean predictive standard deviation decreased from 0.70 to 0.22. Importantly, GSA conducted across the successive GPR fits consistently identified MgOAc and NTP as the principal contributors to output variance. Thus, filtering substantially improved model performance without altering the principal process-level sensitivity conclusion.

### 4.2 Final Experimental-Response Surrogate GPR Model

This section presents results generated from the application of the computational methodology on the mRNA IVT process. To verify that the proposed diagnostic framework accurately captures underlying system physics, the surrogate’s output is benchmarked against established IVT kinetic behavior reported in the literature.

#### 4.2.1 Determination of Statistically Significant Inputs

After data cleaning, the final experimental-based GPR model achieves an *R* ^2^ value of 0.90. The outcomes of GSA analysis conducted on this model are presented in Figure 5.

**Figure 5:**
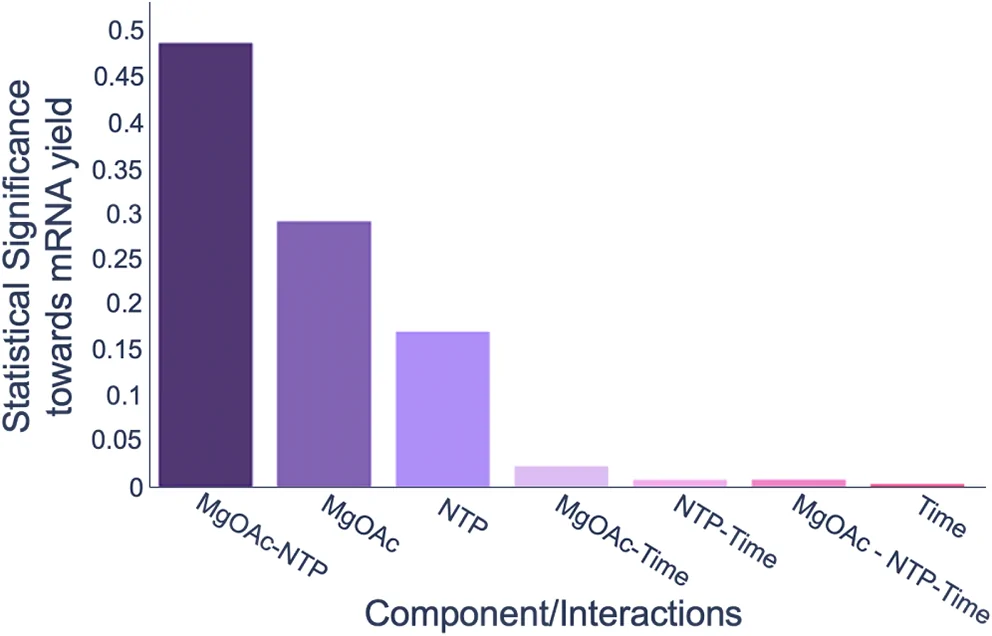
Inputs and their interactions in the experimental-based GPR model.

According to the figure, the results are reasonable from a biological standpoint. First, the dominant effect of free magnesium and NTP complexes is biologically plausible because of their fundamental roles in T7-RNA polymerase-catalyzed nucleotide incorporation during mRNA synthesis. The greater significance of MgOAc than NTP is also biologically plausible, since Mg^2+^ contributes both to the formation of Mg-NTP substrate complexes and to the Mg^2+^-dependent catalytic environment of the T7-RNA polymerase active site. The mechanistic basis for these coupled roles is illustrated in Figure 6.

**Figure 6:**
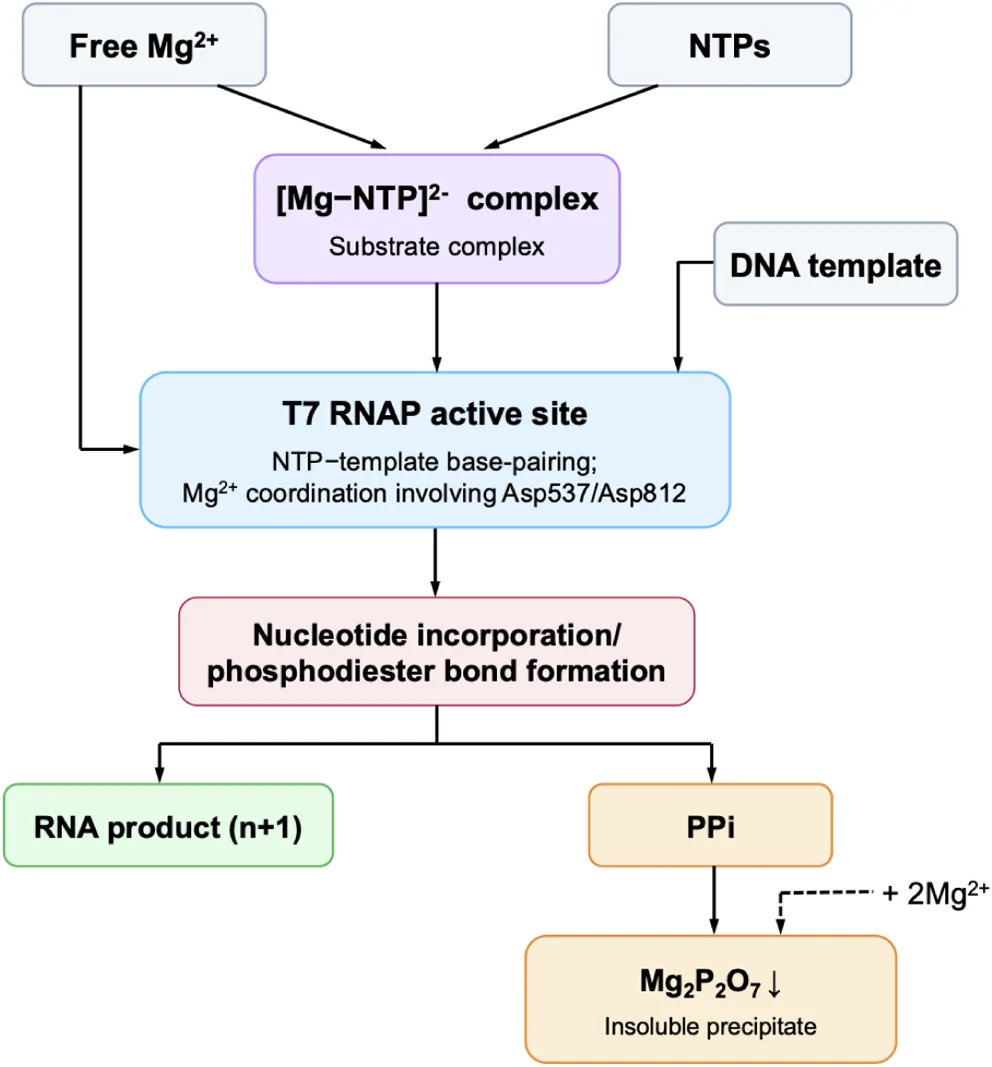
Schematic representation of key mechanistic steps involved in T7 RNA polymerase (T7RNAP)-catalyzed IVT.

Furthermore, although incubation time may be almost statistically negligible on its own, it does play a role as part of binary and 3-way interactions with MgOAc and NTP. This is justifiable as time affects the extent of the IVT reaction.^36^ Specifically, the incubation time examined ranged from 15 min to 4 h, while it is generally understood that at least 1 h is required for IVT completion under typical conditions. Under optimal reaction conditions, however, a substantial fraction of RNA synthesis occurs within the first 15 min, whereas under suboptimal Mg and NTP conditions the reaction rate decreases significantly, requiring longer incubation times to achieve comparable yields. This likely explains the statistical significance observed for the interaction between incubation time, Mg, and NTP concentrations.

Surprisingly, the statistical significance of T7-RNA polymerase is negligible although this enzyme is necessary for IVT to take place. Low enzyme concentrations lead to inefficient transcription and reduced mRNA yields, while excessive amounts may promote side-product formation. However, several factors may explain its limited influence on model fitting. First, although T7 RNA polymerase directly governs transcription kinetics as the catalytic enzyme of the IVT reaction, its contribution to the output variance was smaller relative to MgOAc and NTP concentrations within the experimental design space explored. Second, the polymerase concentration range examined may already lie near a kinetically sufficient or partially saturated regime, reducing its apparent statistical significance in the GSA. Finally, MgOAc and NTP concentrations exert strong direct and interaction effects through Mg–NTP complex formation and substrate availability, thereby dominating the observed variance in mRNA yield.

Template DNA and spermidine exhibited negligible statistical significance toward mRNA yield within the experimental design space explored. Although template DNA directly influences transcription by providing the reaction template, the concentration range investigated may already correspond to a sufficiently nonlimiting regime, thereby reducing its contribution to the observed output variance relative to MgOAc and NTP concentrations. Spermidine, on the other hand, primarily acts as a stabilizing polyamine additive that mitigates inhibitory effects and supports nucleic acid interactions, rather than directly participating in the IVT reaction stoichiometry.

#### 4.2.2 Design Space Determination

Based on the LHS generated samples fed into the experimental-based GPR model, the favorable regions of the design space associated with high mRNA yield can be identified with respect to the statistically significant parameters, namely the concentrations of MgOAc, NTP and incubation time (see Figure 5).

First, the simultaneous effect of MgOAc and NTP concentrations, the most statistically significant inputs, on mRNA yield is presented in Figure 7.

**Figure 7:**
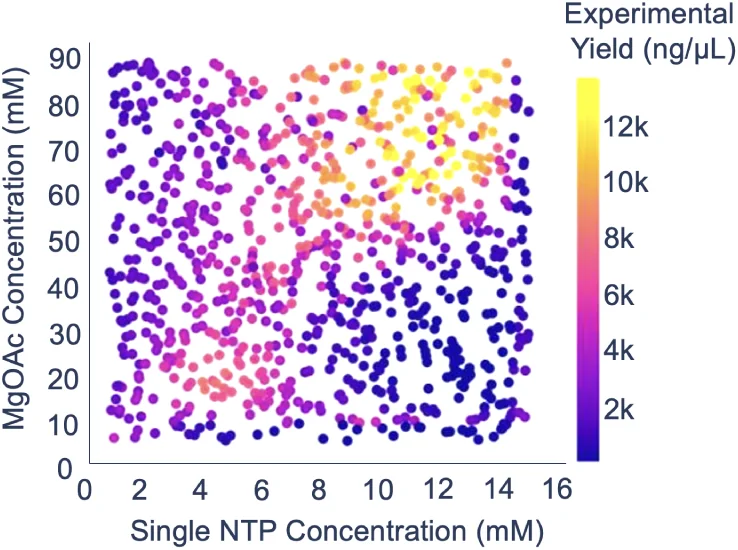
Simultaneous effect of MgOAc and NTP concentration on experimental mRNA yield.

As Figure 7 evidences, the product yield reaches its maximum when both MgOAc and NTP concentrations are simultaneously high. Interestingly, there also exists a region with substantially lower concentrations of these inputs that still produces medium to high mRNA yields. Magnesium ions serve as essential cofactors for RNA polymerase activation, stabilizing the negative charges of the NTP triphosphate groups and making the substrates suitable for enzymatic reaction and binding within the polymerase active site on the DNA template. Most RNA polymerases employ a two-metal-ion catalytic mechanism in which paired Mg^2+^ ions coordinate RNA synthesis. One Mg^2+^ ion facilitates the positioning and binding of the incoming NTP at the catalytic site, while the second Mg^2+^ ion catalyses phosphodiester bond formation by stabilizing the transition state and promoting nucleophilic attack, thereby enabling efficient and accurate RNA synthesis.^2^

In addition to their catalytic role, Mg^2+^ ions contribute to the structural stability of the polymerase–nucleic acid complex by maintaining proper alignment of the RNA/DNA hybrid and supporting transcriptional fidelity. Conversely, suboptimal Mg^2+^ levels can lead to premature termination, reduced polymerase fidelity, and destabilization of transcriptional machinery, sometimes leading to global changes in gene expression patterns. NTP concentration determines the substrate availability for chain elongation, with insufficient levels slowing transcription kinetics and excessive levels potentially causing substrate inhibition or altering enzyme dynamics.^6^ Indeed, points with yields greater than 8000 ng/*mu*L are concentrated within a band consistent with an approximately proportional relationship between MgOAc and NTP concentrations. This suggests that high yields are associated with a particular range of MgOAc-to-NTP ratios, which is investigated further in Figure 8.

**Figure 8:**
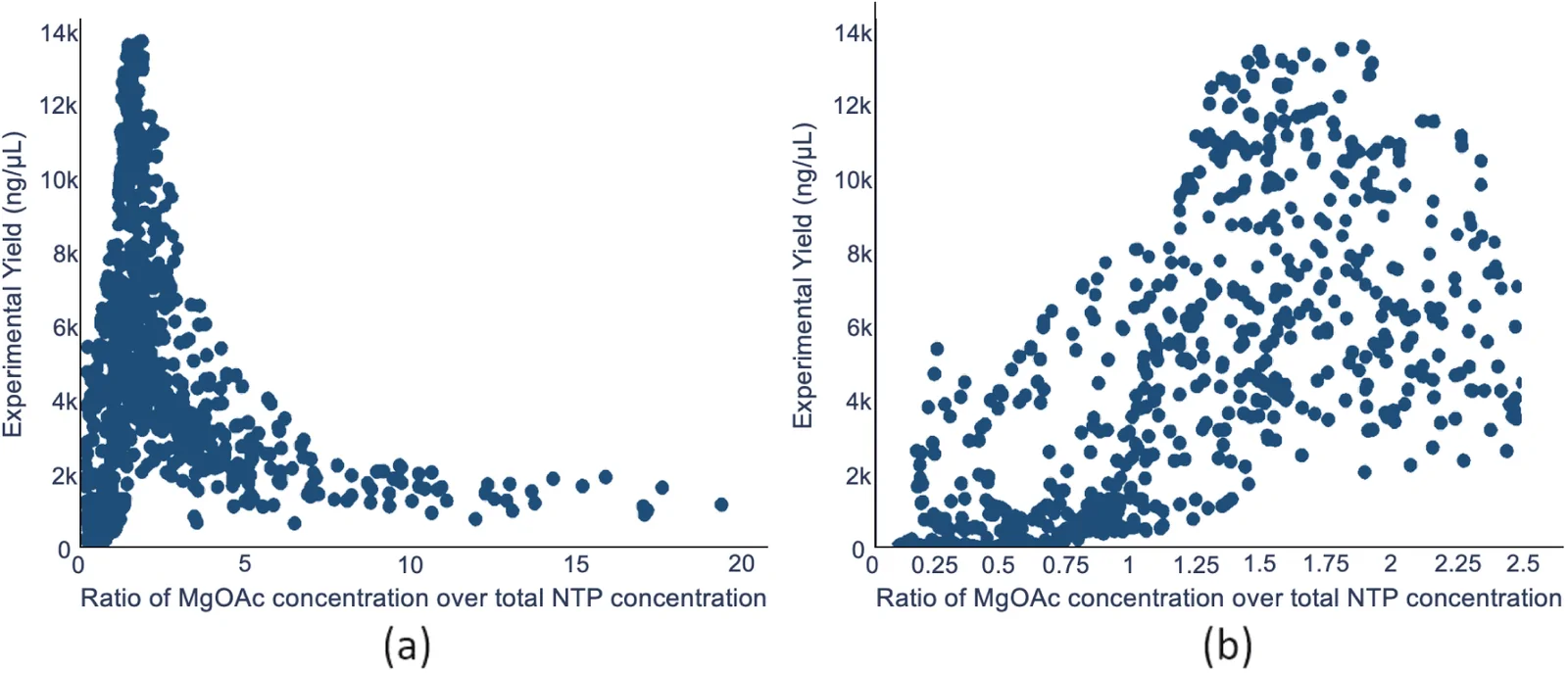
Effect of ratio of MgOAc concentration over total NTP concentration on experimental mRNA yield. (a) Overall investigation across all ratios used for the GPR model. (b) Close up to the ratio range resulting in the greatest mRNA yields.

Figure 8 showcases that the optimal ratio for yield maximization is between 1.25 and 2, in line with previous observations^34,35^ stating an optimal ratio of 1–1.9. This can be explained via the influences of Mg^2+^ to NTP ratio on RNA synthesis efficiency by balancing substrate activation, catalytic function, and enzyme stability. The MgNTP^2–^ complex is the active substrate for elongation in RNA synthesis. Too low Mg^2+^ relative to NTPs leads to insufficient charge neutralization of NTP triphosphates, reduced catalytic activity, poor substrate binding which is responsible for lower RNA yield. Too high Mg^2+^ relative to NTPs can inhibit RNA polymerase activity by increasing ionic strength, causing nonspecific interactions, substrate inhibition, or RNA degradation via hydrolysis, which reduces both yield and transcript quality. While having the optimal Mg^2+^-NTP ratio prevents inhibition effects seen with excess Mg^2+^ or NTPs, the overall reaction remains slow and inefficient because absolute substrate and cofactor amounts are limiting. *In vitro* studies show transcription stalling and poor yield when Mg^2+^ or NTP are below threshold levels, even at optimal ratios.^37^

Finally, although incubation time has a negligible first-order contribution to mRNA-yield variation, the GSA identifies interaction effects between incubation time and the concentrations of MgOAc and NTP. The relationship between incubation time and mRNA yield is therefore further illustrated in Figure 9.

**Figure 9:**
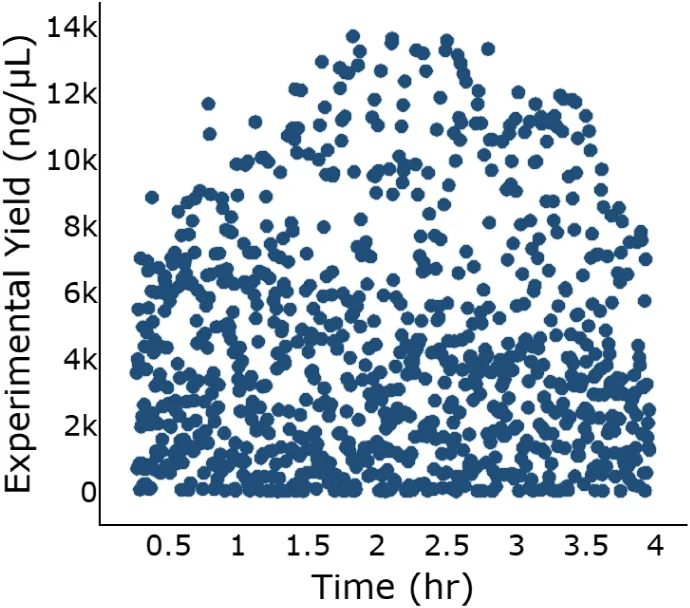
Experimental mRNA yield formation at different incubation times.

As demonstrated in Figure 9, no clear independent trend between incubation time and mRNA yield is evident across the investigated design space. However, within the plotted data, yields above 12,000 ng/*μ*L are observed only at incubation times between approximately 1.5 and 3 h. This weak tendency is consistent with the GSA results, suggesting that the influence of incubation time is conditional on the underlying MgOAc and NTP concentrations rather than through a strong independent effect.

### 4.3 Surrogate Model of Error between Mechanistic Model- and Experimental-Based mRNA Yield Predictions

The GPR surrogate model of error represents the model–experiment discrepancy and has an *R* ^2^ value of 0.86. The GSA conducted on the model led to the results presented in Figure 10a. Furthermore, a GPR model is generated for validation purposes where the only inputs considered are NTP, MgOAc and T7-RNA polymerase, namely the most statistically critical components as showcased in Figure 10a. The GSA outcomes on this validation GPR model are presented in Figure 10a and b.

**Figure 10:**
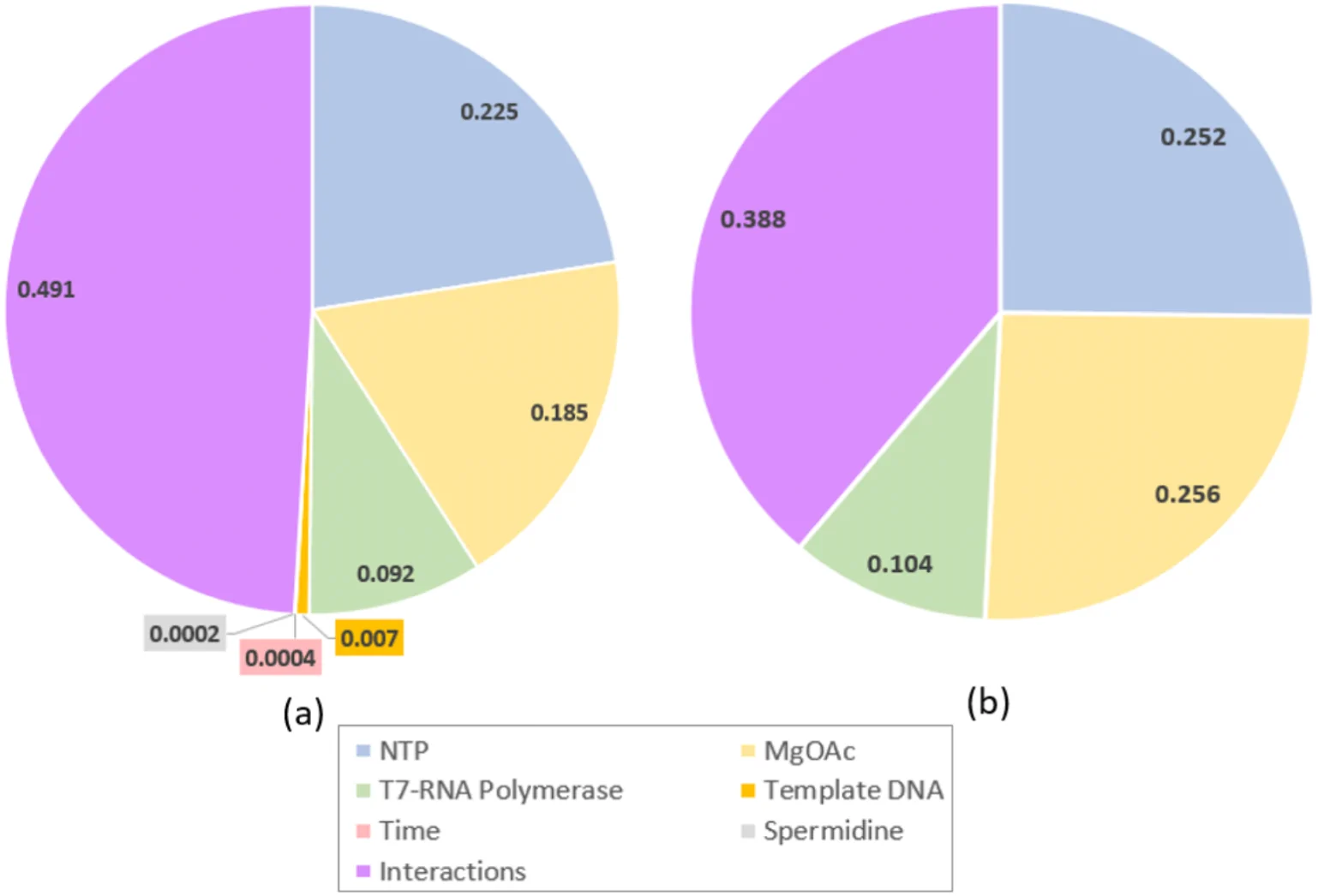
Statistical significance of input parameters based on first-order Sobol indices for the surrogate GPR model of error. (a) GPR model generated with all inputs. (b) GPR model generated for validation purposes, only including NTP, MgOAc and T7-RNA polymerase.

When all inputs are considered (Figure 10a), the statistical significance of the interactions is 0.491 whereas for the validation dataset (Figure 10b), this value is equal to 0.388. In other words, although template DNA, incubation time, and spermidine are negligible to the outcome’s variance, their interactions within the IVT reactor system are statistically important. A further investigation is conducted through the exploration of the closed Sobol indices in conjunction with the first order ones. That way, the key components and interactions that affect the full surrogate model’s output are determined, the outcomes of which are presented in Figure 11.

**Figure 11:**
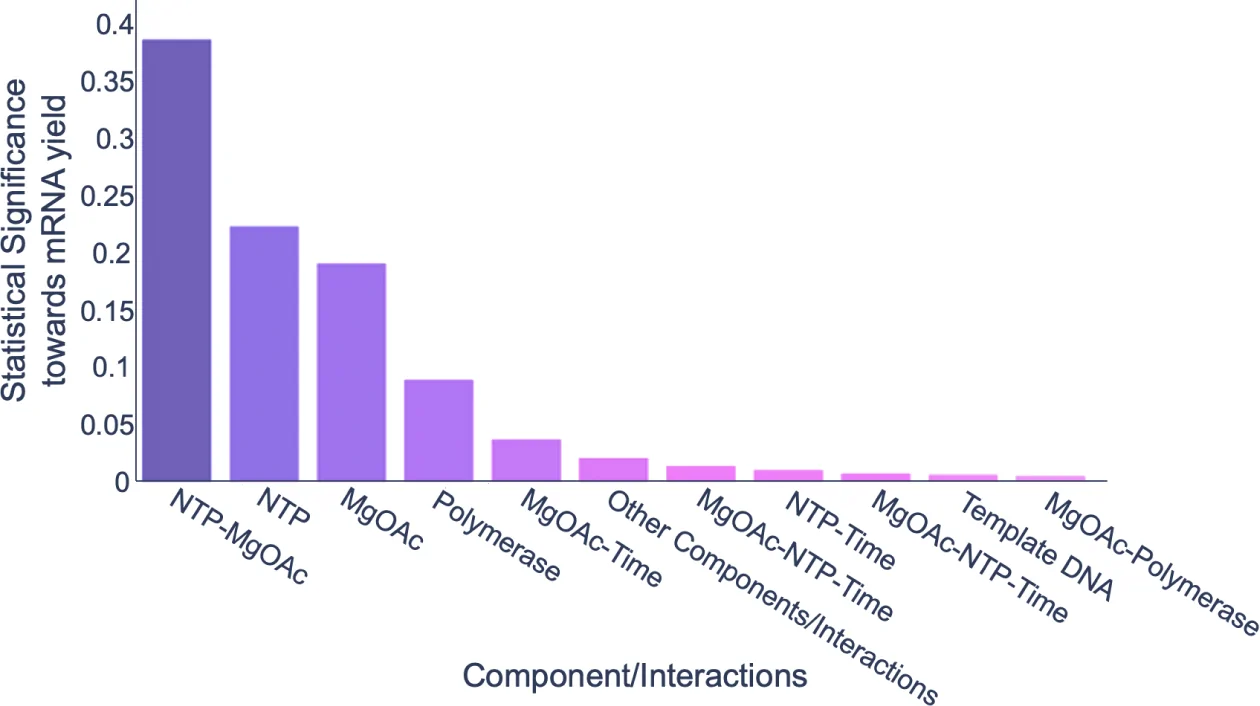
Statistical significance of all input parameters and their interactions on the output variance of the surrogate model of error.

It is evident from Figure 11 that the greatest contributor to the residual between simulation and experimental yield is the binary interaction between NTP and MgOAc followed by the individual NTP and MgOAc effects. T7-RNA polymerase and the binary interaction of MgOAc with time also appear to play a role. Finally, the remaining components and interactions only add up to a total of 0.06. The most significant among these minor contributors are presented separately in the figure.

Importantly and based on Figure 11, to guide subsequent mechanistic investigation, priority should be given to involved differential and algebraic equations^14^ which include NTP and MgOAc or free magnesium ions. Readjusting parameters of differential equations and validating equilibrium constants of chemical reactions represent potential subsequent refinement actions. Moreover, since T7-RNA polymerase as an individual component contributes to the error between simulation and experimental yields, the consumption rate constant of pure T7-RNA polymerase should also be prioritized for further investigation. Furthermore, variability in enzyme quality, concentration, or kinetics may not be fully captured by mechanistic models often assuming ideal enzyme kinetics and saturation conditions. However, in practical experiments, batch-to-batch enzyme variability, degradation, or suboptimal activity can impact yields. The surrogate error model is sensitive to such variability, highlighting T7 RNA polymerase as a significant factor affecting discrepancies from theoretical predictions. Lastly, given that the next most statistically significant contributor to the output is the binary interaction of MgOAc with time, the observed discrepancy may be associated with the rate of consumption of free magnesium ions as well as the differential expression for its inhibitory precipitation.

### 4.4 Focus on Mechanistic Model Kinetic Parameters

The results of the GSA conducted on the GPR model with the kinetic parameters as inputs are presented in Figure 12. These results are purely model-based, hence it is important to compare them critically with the surrogate model of error to identify which mechanistic parameters and associated model components should be prioritized for further investigation.

**Figure 12:**
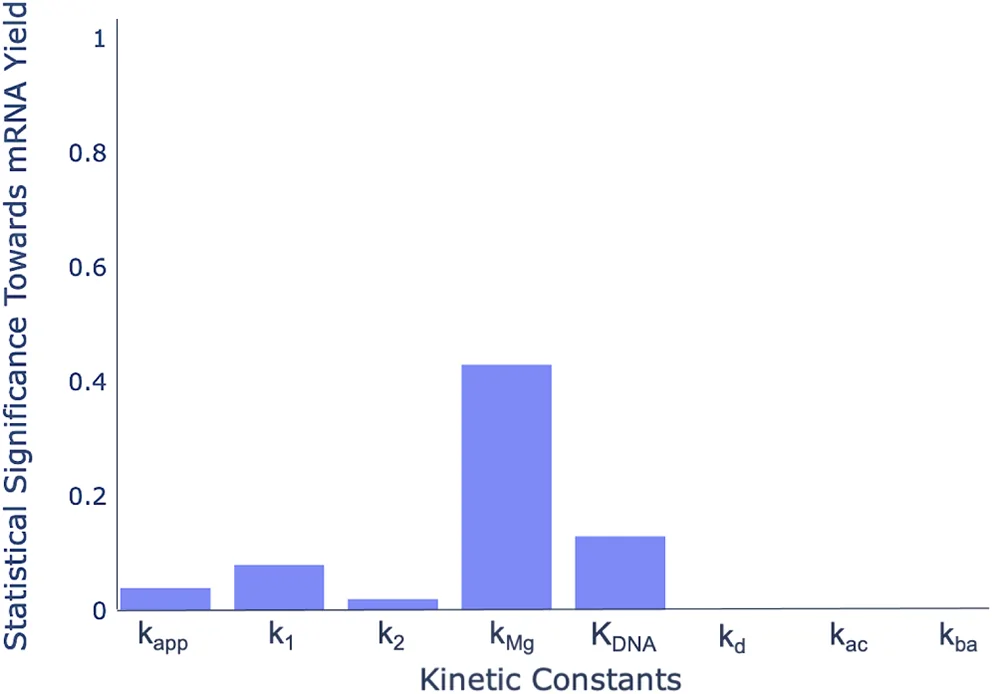
Statistical significance of all kinetic parameters toward mRNA yield as obtained from the first order Sobol indices.

From Figure 12, the four main constants of statistical significance in descending order are the Mg degradation rate constant (*k*_*Mg*_), the DNA equilibrium constant (*K*_*DNA*_), the Mg saturation constant (*k*_1_) and the transcription rate constant (*k*_*app*_). These results are in line with the results obtained in Section 4.3 about the mechanistic model accuracy. More specifically, the statistically significant components resulting in the residual measured in the surrogate model of error are the NTP, Mg and T7-RNA polymerase. Constants *k*_*Mg*_, *k*_1_ and *k*_*app*_ are the tunable elements of mathematical expressions including NTP, Mg and T7-RNA polymerase in ref 14. Furthermore, the equilibrium constant *K*_*DNA*_ is in a relationship that includes the polymerase concentration.

In summary, the diagnostic evaluations in Sections 4.3 and suggest that kinetic terms involving Mg, NTP, and T7-RNA polymerase represent the primary loci of sensitivity and potential structural discrepancy in the baseline model.^14^ These computational insights offer targeted hypotheses for future investigation.

## 5 Conclusions

The methodology developed in this study sheds light on complex biochemical mechanisms in addition to facilitating the integrated investigation of experimental behavior and mechanistic model performance through better understanding of the process. In this study, we focus on the mechanism of *in vitro* transcription of mRNA. The methodology is based on a logical series of GPR fitted models followed by GSA every time. After GSA is conducted on every GPR model, conclusions are drawn based on the biochemical understanding of the process which lead to the following GPR fitting.

Thanks to this methodology applied to mRNA production, MgOAc and NTP concentrations were identified as the principal contributors to variation in mRNA yield, while incubation time contributed to a lesser extent primarily through interactions with these variables. Within the investigated design space, high mRNA yields were associated with simultaneous MgOAc and individual NTP concentrations of approximately 60–90 mM and 9–14 mM, respectively, and with MgOAc-to-total-NTP ratios of approximately 1.25–2. These ranges therefore represent favorable operating regions rather than formally determined optima. Although no clear independent optimum was identified for incubation time, the highest observed yields occurred at incubation times of approximately 1.5–3 h. Moreover, conditions associated with statistically anomalous observations are prioritized for repeat or targeted experimentation, which can help distinguish experimental variability or error from reproducible process behavior.

Then, through the surrogate model of error as well as the examination of the subsequent mechanistic model’s kinetic parameters, candidate mechanistic model components for further investigation are prioritized. The critical inputs leading to yield variation between the simulation results and the experimental-based GPR model are the NTP, MgOAc and T7-RNA polymerase. This is further supported by the consistency of these findings with the kinetic constant-focused GPR model. That way, kinetic parameters and associated model components that should be prioritized for subsequent parameter recalibration or structural investigation are identified.

The present study demonstrates the analytical and prioritization stages of the framework, establishing how experimental response information, model–experiment discrepancy and mechanistic parameter sensitivity can be integrated to direct subsequent investigation. The resulting priorities provide clear feedback routes for repeat or targeted experimentation, parameter recalibration and structural model refinement, as illustrated in Figure 3. These corrective actions are not implemented within the present case study; completing this subsequent intervention and reassessment cycle would provide an important next demonstration of the framework.

Although demonstrated here for a single IVT case study, the structure of the framework is not specific to IVT and is formulated around information common to experimental-mechanistic modeling problems: experimental observations, surrogate representations, model–experiment discrepancy and mechanistic parameter sensitivities. The present results nevertheless demonstrate its application within the experimental dataset, operating ranges and mechanistic model considered here. Future work should therefore evaluate the framework using independent datasets, alternative operating ranges and mechanistic representations, as well as other biochemical or chemical systems, to further establish its robustness and transferability. Within IVT itself, the framework could also be extended to alternative response variables associated with product quality, such as mRNA integrity or double-stranded RNA formation.
